# Chances to Have A Boy after Gender Selection by Pre-Implantation Genetic Screening Are Reduced in Couples with only Girls and without A Boy Sired by The Male Partner

**DOI:** 10.22074/ijfs.2016.4828

**Published:** 2016-11-01

**Authors:** Soryya Panahi, Fariba Fahami, Mohammad Reza Deemeh, Marziyeh Tavalaee, Hamid Gourabi, Mohammad Hossain Nasr-Esfahani

**Affiliations:** 1 Department of Reproductive Biotechnology, Reproductive Biomedicine Research Center, Royan Institute for Biotechnology, ACECR, Isfahan, Iran; 2 Department of Midwifery, School of Nursing and Midwifery Care Research Center, Isfahan University of Medical Sciences, Isfahan, Iran; 3 Department of Genetics, Reproductive Biomedicine Research Center, Royan Institute for Reproductive Biomedicine, ACECR, Tehran, Iran; 4 Isfahan Fertility and Infertility Center, Isfahan, Iran

**Keywords:** Pre-Implantation Genetic Screening, Pregnancy, Implantation, Abortion

## Abstract

**Background:**

Gender selection and family planning have their roots in human history. Despite great interest in these fields, very few scientific propositions exist which could explain why some family do not attain the desired sex. Therefore, the aim of this study was to evaluate whether sex of previous child or children could affect the outcomes of pre-implantation genetic screening (PGS).

**Materials and Methods:**

This historical cohort study including 218 PGS cases referring to Isfahan Fertility and Infertility Center (IFIC). Couples were grouped as those who their male child passed away or her husbands’ has a son(s) from their previous marriage (n=70) and couples who just have daughter (n=148). Male normal blastocysts were transferred for both groups. The outcomes of PGS including pregnancy, implantation and abortion rates, along with possible confounding factors were compared between the two groups.

**Results:**

Significant differences in pregnancy, implantation and abortion rates were observed between couples whose their male partner had/has one boy (n=70) compared to those who have just girl(s) (n=148) despite similar number and quality of male normal blastocyst transferred in the two groups. Confounding factors were also considered.

**Conclusion:**

The Ybearing spermatozoa in male partners with no history of previous boy have lower ability to support a normal development to term, compared to male partners with previous history of boy requesting family balancing.

## Introduction

Gender selection has its roots in ancient culture and this desire has even lasted through this millennium. The alternative name for this desire is family balancing (1). Beside the cultural aspects of gender selection, communal pressures, ethnicity, social economical and educational status of couples influence parental procreative desire. In some society, urge for such a desire has led to illegal abortion of undesired sex, family pressures and even divorce (2). Considering the fact that parental procreative desire for boy is much higher than girls, in some rural communities, couples may continue their fertility, despite having several girls, in hope of a boy delivery. Therefore, there appear to be an obligation for these couples to bear a male heir for the family. In these communities, sometime they may name their girls as: “die di” in Hong Kong and China meaning “bring a younger brother” (3), “ghez bas” in Turkish meaning the last daughter.

Despite ancient history of gender selection, the mechanism or etiology of gender determinant in natural conception cycle remains unknown and except some equivocal hypothetical proposition, there is no solid theory, except random opportunity for X or Y bearing sperm penetrating an oocyte (4).

Iran, with high ethnic backgrounds (Fars, Lors, Kords, Turks, Arabes, Blooch and Turkamen), some families feel obliged to bear a male heir for the family. Therefore, gender selection technology, especially pre-implantation genetic screening (PGS) which has high accuracy, has provided an opportunity for these couples to fulfill this procreative paternal desire for family balancing.

Gender selection in this era has opened a hot ethical issue in different societies. Critics believe that family balancing is the right of all parents with procreative desire for certain sex (5). Therefore, based on recent epidemiological studies which show reduced general fertility rate in Iran (6) and furthermore, revealing that a small percentage of community may opt for gender selection procedure like PGS, are among the reasons why this procedure, as family balancing method, is provided by some infertility centers. Therefore, regardless of ethical issue of gender selection, which is out of scope of this study, the aim of this historical cohort study is to evaluate whether sex of previous child or children, could affect the outcomes of PGS. Therefore, the male partner of the couples were grouped to those who had/has one boy or those who have just girls.

## Materials and Methods

This is a historical cohort study including 218 PGS cases based on below inclusion and exclusion criteria referring to Isfahan Fertility and Infertility Center (IFIC). In this center, couples are routinely informed and written consent are obtained to allow the usage of their data for research purposes. The study was approved by the Ethical Committee of IFIC.

### Inclusion criteria

Couples who requested PGS for male gender selection for family balancing were included in this study. Therefore, PGS was carried out for the couples who had at least two children of the opposite sex, in this circumstance at least two girls and/or couples with history of having male (their son died and have two girl or have a son from their first marriage and were seeking from family balancing for their second marriage with two girls).

### Exclusion criteria

i. Female with age higher than 42 years, ii. History of medical or obstetric complications within previous pregnancies, including premature delivery, low birth weight, stillbirth, gestational hypertension, gestational diabetes and abnormal uterine, iii. Maternal systemic diseases (cardiovascular, renal, glands disease, cancer, etc) that have adverse impact on pregnancy, iv. Previous history of infertility, v. history of habitual abortion (> 2 abortions), vi. candidates of family balancing, with at least two girls, who did not undergo embryo transfer due to ovarian hyper stimulation syndrome or unsuitable endometrium (grade C or endometrium with less than 6 mm thickness), and vii. Couples who had no normal male blastocyst for transfer.

### Ovulation induction, intra cytoplasmic sperm injection and pre-implantation genetic diagnostic

Briefly, following ultrasound scan on day 2 of menstrual cycle, patients underwent ovarian stimulation with gonadotropins and gonadotropin-releasing hormone (GnRH)-antagonist following serial monitoring. Human chorionic gonadotropin (HCG) was administered when at least two leading follicles measured 18 mm in diameter were observed. 36 hours later transvaginal ultrasound-guided oocyte retrieval was performed.

Intra cytoplasmic sperm injection (ICSI) was implemented for all the cases and embryo biopsy for PGS procedure performed on day 3 following oocyte retrieval by direct aspiration of a single blastomere through an opening created by Laser (Hamilton Thorne, New Zealand) in the zona pellucida. Following fixation of the biopsied blastomere, nuclear DNA was analyzed by Fluorescent In Situ Hybridization (FISH) method for 3 chromosomes (X, Y, and 18). Embryos were scored on day 5 and stage of development were recorded (arrested, compact, early blastocyst, expanded blastocyst and hatched blastocyst) (7). One or maximum of 2 male blastocyst(s) were transferred per patients. Percentages of chemical and clinical pregnancies, implantation and abortion rates were assessed according to Deemeh et al. (8).

Variables analyzed include female and male age, semen parameters, number of retrieved oocytes, number of oocytes injected, fertilization rate, number of embryos biopsied, number of blastocysts transferred or frozen, and the fate of the remaining embryos.

Based on X, Y and 18 chromosomes, percentages of eligible male and female embryos, male and female blastocyst were assessed. In addition, percentages of eligible and abnormal male and female arrested embryos, and, not founded or not diagnosed embryos were determined.

In order to facilitate the calculation, number of PGS embryos whose biopsied blastomere had no nuclei or signals, were deducted from the number of PGS embryos. Therefore, the number of PGS embryos refers to embryos that were biopsied and had a genetic report regarding chromosomes X, Y and 18. Furthermore, in this study “normal” refers to embryos that had correct set of chromosomes follow screening for X, Y and 18.

### Patient categorization

Couples seeking family balancing in the IFIC were categorized to two groups: i. Those with history of having male progeny and ii. Those that have at least two girls. Considering the limited number of couples in the first group, in order to reduce confounding effect, the couples for the second group were chosen within the period that the couples from the first group were performing PGS.

### Statistical analysis

Statistical analyses were performed using Chi square and independent t test using SPSS version 16. P<0.05 was considered as statistically significant.

## Results

Table 1 shows the clinical outcomes of normal male embryo screen by PGS between couples with history of male partner having a previous boy and couples who have girls when requesting PGS for family balancing. The chemical pregnancy (60.0 vs. 16.9%), clinical pregnancy (58.6 vs. 16.9%) and ongoing pregnancy (57.1 vs. 6.1%) rates were significantly different between the two groups. Similarly, implantations (46.9 vs.14.97%) and abortion rates (7.14 vs. 64.0%) were significantly different between the two groups.

**Table 1:** Comparison of clinical outcomes of normal male embryo screen by pre-implantation genetic screening (PGS) between couples with and without history of previous boy


Clinical outcomes	No history of previous boy	History of previous boy	P value

Chemical pregnancy	16.9%	60.0%	<0.001
	(25/148)	(42/70)	
Clinical pregnancy	16.9%	58.6%	<0.001
	(25/148)	(41/70 )	
Ongoing pregnancy	6.1%	57.1%	<0.001
	(9/148)	(40/70)	
Non-pregnant	83.1%	40.00%	<0.001
	(123/148)	(28/70)	
Abortion rate	64.00%	7.14%	<0.001
	(16/25)	(1/41)	
Implantation rate	14.97%	46.9 %	<0.001


Following this analysis, in order to evaluate the differences observed between the two groups were not due to difference in parameters related to embryos quantity and quality (Table 2), and also other confounding parameters (Tables3, 4), these parameters were compared between the two groups. The number of retrieved oocyte (11.1 ± 5.4 vs.10.9 ± 4.5) and injected oocyte (8.2 ± 3.5 vs. 8.4 ± 3.1) were similar between two groups and no significant differences were observed. However, a statistically significant difference (P<0.05) was found in the number of immature oocytes between couples without previous history of boy (7.4 ± 11.4) and couples with previous history of boy (4.2 ± 7.72). Percentage of fertilization also were similar between two groups (75.3 ± 17.6 vs. 78.6 ± 18.1). When the same analysis was performed for embryonic parameters, we observed that the number of PGD embryos (5.9 ± 3.0 vs. 6.0 ± 2.7), number of embryos not found (0.5 ± 0.8 vs. 0.7 ± 1.0) and total blastocyst (4.1 ± 2.1 vs. 4.4 ± 2.2) were similar between the groups studied and there was no statistically significant difference. However a statistically significant difference (P<0.05) was observed in the number arrested embryos (1.3 ± 1.3 vs. 0.9 ± 1.1) between two groups. But, percentage of this parameter was not statistically significant (Table 2).

In addition, we compared normal and abnormal embryonic parameters based on X, Y and chromosome 18 analyses in the couples with and without previous history of boy. We did not observed any significant difference in male and female embryos based on day 3 report, male and female blastocyst, and also male and female embryos arrested between two groups except abnormal female blastocyst and normal female embryos arrested (Table 3).

**Table 2:** Description of oocyte, fertilization and embryonic parameters in the groups with and without pervious history of boy


	Parameters	No history of previous boy	History of previous boy	P value

Oocyte	Retrieved	(11.1 ± 5.4)	(10. 9 ± 4.5)	0.7
	Injected	(8.2 ± 3.5)	(8.4 ± 3.1)	0.6
	Immature	7.4 ± 11.4	4.2 ± 7.72	0.01^*^
	Fertilization rate	75.3 ± 17.6	78.6 ± 18.1	0.2
Embryonic parameters	PGS embryos	100%	100%	0. 8
		(5.9 ± 3.0)	(6.0±2.7)	
	Arrested embryos	20.5 ± 18.1	15.1 ± 19.0	0.07
		(1.3 ± 1.3)	(0.9 ± 1.1)	(0.03)^*^
	Not found	8.0 ± 12.6	11.4 ± 15.7	0.09
		(0.5 ± 0.8)	(0.7 ± 1.0)	(0.07)
	Total blastocyst	72.7 ± 18.7	74.2 ± 20.3	0.6
		(4.1 ± 2.1)	(4.4 ± 2.2)	(0.4)


Percentages are presented outsides the parenthesis and numbers in the parenthesis. ^*^; P Value less than 0.05 statistically significantly and PGS; Pre-implantation genetic screening.

**Table 3:** Description of embryonic parameters based on X, Y and chromosome 18 analyses in the group with and without previous history of boy


	Parameters	Statues	No history of previous boy	History of previous boy	P value

Based on X, Y and 18	Male embryos based on day 3 report	Normal	40.9	40.4	0.9
			(1.9 ± 1.1)	(2.02 ± 1.2)	(0.6)
		Abnormal	10.0 ± 13.1	9.6 ± 16.9	0.8
			(0.6 ± 0.9)	(0.5 ± 0.9)	(0.3)
	Female embryos based on day 3 report	Normal	29.7 ± 22.4	26.8 ± 21.0	0.4
			(1.7 ± 1.6)	(1.5 ± 1.8)	(0.4)
		Abnormal	19.5 ± 19.4	23.2 ± 20.5	0.2
			(1.1 ± 1.1)	(1.2 ± 1.1)	(0.4)
	Male blastocyst	Normal	38.9 ± 21.1	39.2 ±18.4	0.9
			(1.8 ± 0.9)	(2.0 ± 1.2)	(0.3)
		Abnormal	5.9 ± 10.3	6.3 ± 12.1	0.8
			(0.4 ± 0.6)	(0.3 ± 1.0 )	(0.6)
	Female blastocyst	Normal	26.0 ± 21.8	25.4 ± 20.7	0.8
			(1.5 ± 1.5)	(1.4 ± 1.4 )	(0.7)
		Abnormal	8.5 ± 12.9	13.4 ± 16.3	0.03^*^
			(0.4 ± 0.7)	(0.7 ± 0.8 )	(0.02)^*^
	Male embryos arrested	Normal	2.0 ± 6.0%	1.2 ± 5.2	0.4
			( 0.1 ± 0.4)	(0.1 ± 0.2)	(0.06)
		Abnormal	4.0 ± 8.3%	3.2 ± 10.3	0.6
			(0.3 ± 0.6)	(0.2 ± 0.6)	(0.3)
	Female embryos arrested	Normal	3.6 ± 8.4%	1.4 ± 5.4	0.02^*^
			(0.2 ± 0.5)	(0.1 ± 0.4)	(0.04)^*^
		Abnormal	10.9 ± 13.8%	9.8 ± 15.4	0.6
			(0.6 ± 0.7 )	(0.5 ± 0.7)	(0.4)


Percentages are presented outsides the parenthesis and numbers within the parenthesis. ^*^; P value less than 0.05 statistically significant.

The same analysis was performed on male and female characteristics in the couples with and without previous history of boy. Female and male age, percentage of sperm motility, normal morphology, sperm concentration and number of previous spontaneous abortion were similar between two groups (Table 4).

Qualities of blastocysts were assessed according to Gardener criteria (7) and no significant difference was observed between the qualities of blastocysts transferred between the two groups (data not shown).

**Table 4:** Comparison of male and female characteristics in the group with and without previous history of boy


Parameters	No boys	One or more boy (s)	P value

Female age (Y)	34.7 ± 4.00	34.3 ± 4.4	0.5
Male age (Y)	40.4 ± 4.8	40.3 ± 5	0.9
Number of previous spontaneous abortion	15	2	0.06
Sperm motility (%)	47.0 ± 13.7	46.5 ± 12.5	0. 8
Normal sperm morphology (%)	92.3 ± 5.3	91.8 ± 6.4	0.5
Sperm concentration (10^6^/ml)	55.0 ± 22.5	58.2 ± 23.7	0.4


## Discussion

Importance of gender has its root in history and different reasons have been proposed for why some individuals give birth to only male or female offspring. However, scientist believe that since equal number of boys and girls are born in a society, this can be attributed to equal number of X-and Y-bearing spermatozoa produced during spermatogenesis and their random chance of fertilization. Therefore, the allocation of sex is depended on which X-or Y-bearing spermatozoa reaches the egg first (4). Withstanding this theory, some scientists believe that different factors, like nutrition, resource availability (famine), may skew the random chance of fertilization of the X-and Y-bearing spermatozoa (9, 10). To extend our understanding of the factors which may distort the random chance of fertilization and development of the X-and Y-bearing spermatozoa, we assessed the outcome of PGS sex selection in two groups of couples: those in which the male partner had/has a history of a male and those couples that had/have just female offspring.

The results revealed significant difference in terms of implantation, chemical, clinical and ongoing pregnancy rates between the two groups and these parameters were significantly higher in the group that their male partner had/has a previous boy. The rate of abortion was also substantially higher in the group that their male partner had no previous boy compared to the other group. Considering similar number or percentage of normal male blastocysts transferred in the two groups, our results suggest that it is likely that the Ybearing spermatozoa have lower ability to support a normal development to term in couples whom their male partner had no previous boy. In order to solidify this possibility by ruling out the confounding factors, we compared both mean number and/or percentage of the various factors between the two groups. Among these factors only total number of immature oocytes, number of embryos with normal female that arrested (did not developed to blastocyst), and percentage of abnormal female embryos that did reach blastocyst stage were significantly different between the groups.

Number of immature oocyte were higher in the group with no previous boy, although this factor could have confounding effect but considering the fact that other factors like number of normal male blastocysts were similar in both groups, we believe the influence of this factor could not be substantial. If the number or percentage of female arrested embryos were higher in the group with history of previous boy, this is would be against our conclusion but since this parameter is higher in the other group, logically this does not affect our conclusion. Higher number of normal female embryo that arrested in the group with history of previous boy compared to the other group, suggest that one of the reasons for higher chance of conception for boy in this group might be related to this factor but this possibility does not rule out lower chance of implantation and pregnancy in the group with no history of boy since they have similar number of normal male blastocyst transferred between the two groups.

Therefore, based on the above evidence, we concluded that chance of a Y-bearing sperm to support normal development to birth in the couples with previous history of girl and no boy is reduced and this proposition may be considered as one but not the sole reason why in some couples attempts to have a boy baby is reduced. Although, historically, such a hypothesis may have been theoretically proposed at scientific level, but to the best of our knowledge no scientific data has been published or provided on this subject. Study of literature on this context suggest that in some species meiotic derivers and suppressors especially those related to sex-linked meiotic drive may skew in sex ratio or induce phenotype like “hybrid male sterility”. Recent report by Soh et al. (11) suggest that male-specific region of the Y (MSY) chromosome, with massively amplified gene families may have role in this process. Indeed, it has been shown that “mice Knock-down of Sly or Slx, one of the three X-Y gene pairs, also distorts sex ratio in favor of females or males, respectively”. These authors state that “the mouse MSY’s three acquired and massively amplified gene families and their X homologs are reminiscent of a meiotic driver and suppressor pair: in all three cases, both the X and Y genes are highly amplified, they are expressed specifically in testicular germ cells, and perturbation of gene family copy number results in sex ratio distortion”. However, a long way remains ahead of researchers in this filed to evaluate whether the observation in this study may be related to massively amplified gene families which can act as repressor or drivers and may skew the sex ratio.

Study of literature based on animal studies and hypothetical propositions suggest that mothers may have some impudence over the sex of their offspring and factors like increased follicular testosterone, presence of glucose in the uterine environment and female testosterone levels rise in response to environmental stressors may skew the sex ratio (12-15). Furthermore, study of literature show that in stress condition, pregnant women disproportionately aborts male fetus (16). Although, we did not study these factors in our population but could higher stress condition in the couples who had no history of previous boy, may have influenced their implantation and pregnancy rates? This possibility remains to be determined.

One of the underlying mechanisms which may explain our proposed theory might be the difference in degree of DNA fragmentation in Xand Y-bearing sperm in individuals with no history of previous boy. Although we were not able to assess the degree of DNA fragmentation in Xand Y bearing sperm, but previous study suggests introducing sperm to stress condition, like heat stress, the chance of survival of their X-bearing sperm is higher than the Y-bearing sperm (17). Considering the fact that in our study the barriers of fertilization is bypassed by ICSI, the chance of normal male blastocyst formation is equal between the two groups, but their post blastocyst development is reduced in group with no history of previous boy. This is a hypothetical proposition and need future validation.

Another possible mechanism which may explain our observation in this study might be the bias selection or response of endometrium between the two groups of patients, despite similar number and quality of normal boy blastocysts transferred between the two groups. In line with such a possibility a recent study showed differential gene expression to Y-and X-bearing sperm population inseminated into uterine of porcine uterus (4).

## Conclusion

To our knowledge this is the first report, concluding that chance of pregnancy to term is significantly higher when male normal blastocyst are transferred to couples whose male partner had/has a boy compared to couples whose male partner have just girls.
